# Acetylcholinesterase activity in the brain of alloxan diabetic albino rats: Presence of an inhibitor of this enzyme activity in the cerebral extract

**DOI:** 10.4103/0973-3930.57350

**Published:** 2009

**Authors:** Nayeemunnisa Ahmed, Suraiya Tarannum

**Affiliations:** Department of Pharmacology, Al-Ameen College of Pharmacy, Hosur Road, Bangalore-560 027, India; 1 Department of Telecommunications Engineering, AMC College of Engineering, Bannerghatta Road, Bangalore- 560 083, India

**Keywords:** AChE, alloxan diabetes, brain, inhibitor, cerebral extract, Cichorium intybus (Chicory)

## Abstract

**Background and Aim:**

Ischemic manifestations and cerebral dysfunction have been demonstrated in diabetes. However, the pathogenesis of diabetes-induced cerebral dysfunction still remains to be elucidated. Hence, the present study was initiated.

**Materials and Methods:**

Type-2 diabetes was induced in albino rats (280–300g) with alloxan monohydrate (40 mg/Kg i.v.,) and the cerebrum, cerebellum and medulla oblongata of the brain were used 48 h after alloxan injection for modulations in acetylcholinesterase (AChE, EC 3.1.1.7) activity.

**Results:**

AChE activity in the discrete regions of the brain of rats decreased significantly (P<0.01, 0.05 and 0.05 respectively) in diabetes. In vitro studies using cerebral extract from alloxan diabetic rats demonstrated significant (*P*<0.05) inhibition of AChE activity in the brain of normal animals. Feeding with Cichorium intybus (chicory) leaf extract (500 mg/Kg) for 10 days resulted in an increase in AChE activity.

**Conclusion:**

The impairment in the glycemic control is the basic mechanism causing inhibition of neuronal activity. Cerebral extract from alloxan diabetic rats significantly inhibited the brain AChE activity of normal animals, indicating the presence of an inhibiting factor in the cerebrum of diabetic rats. Cichorium intybus when fed for 10 days offered neuroprotection by stimulating AChE activity.

## Introduction

Diabetes mellitus is a metabolic disorder with a potential to cause cerebral injury.[1,2] In diabetic neuropathy, it is known that cerebral uptake of glucose is significantly affected[3–5] and the brain suffers from hypoglycemic episodes.[6] Diabetes-induced hyperglycemia is known to increase the extent of neurological disorders due to inhibition of enzymatic activities connected to neurotransmission[7,8] in the CNS of mammals and the changes observed have been attributed to alterations occurring in the levels of RNA and proteins.[9] Although the ischemic manifestations and cerebral disorders in diabetes have been documented, the pathogenesis of diabetes-induced cerebral dysfunction still remains to be elucidated. In view of this, the present study was initiated to demonstrate cerebral manifestations of diabetes as in diabetes brain suffers from recurrent episodes of hypoglycemia and impaired glucose oxidation leading to impaired neuronal activity. Efforts have also been made to investigate the neuroprotection offered by *Cichorium intybus* (Chicory) in view of its cardioprotective[10] effects reported earlier.

## Materials and Methods

Male albino rats of Wistar strain (280–300 g) were maintained in polypropylene cages (3 in each cage at 26°C; 12 h light:dark cycle). The animals were fed standard pellet diet and water *ad libitum*. The rats were rendered diabetic with alloxan monohydrate (40 mg/kg; i.v.) and used 48 h after alloxanization. Animals with 220 mg/100 ml blood sugar level were considered diabetic and utilized for experimentation.

The study protocol was approved by the Institutional Animal Ethics committee.

The rats were divided into the following groups:

Gr I: Controls

Gr II: Diabetic

Gr III: Diabetic rats fed on dried leaf powder of *Cichorium intybus* (500 mg/kg) for 10 days.

The animals were killed by decapitation and the brains were quickly removed and washed in ice-cold saline. Different regions of the brain were separated with sterilized fine bent forceps and scalpel, weighed in an electric balance in mammalian Ringer and immediately used for the determination of acetylcholinesterase (AChE) activity.

*Chemicals and reagents*: Alloxan monohydrate was obtained from Sigma Chemicals (St. Louis, MO, USA). All other chemicals used were of analytical grade. Double distilled water was used for biochemical assays.

Homogenate (10% w/v) of different regions of the brain was prepared in ice-cold 0.02 M phosphate buffer (pH 7). The homogenate was centrifuged for 20 min at 6000 rpm and the supernatant was used for the assay. Activity levels of AChE were determined spectrophotometrically by the method of Hestrin.[11]

Effects of *in vitro* administration of the aqueous cerebral extract (0.5 ml) from alloxan diabetic rats on the activity level of brain AChE of normal animal were studied. It was noted from preliminary experiments that only the cerebral extract from the diabetic animal was capable of causing significant decrease in enzyme activity of brain in normal animals and therefore the effects of *in vitro* administration of the extracts from cerebellum, medulla and whole brain of diabetic animals were not studied.

The assay systems, on the addition of aqueous extract of the brain from normal animals constituted controls. Preliminary experiments indicated negligible difference between the activity levels of controls prepared by the addition of cerebral extract from diabetic animals to the assay system after terminating the enzyme reaction and those receiving the extract of the brain from normal animals. Hence, the latter was used as the experimental controls. The assay systems with the aqueous cerebral extract from the diabetic rat were the experimental samples.

### Studies on neuroprotection by intervention with *Cichorium intybus*

#### Cichorium intybus:

(Kasni-Hindi; Hindba Arabic and Chicory-English. The plant has utility in Indian (Unani) system of medicine for the maintenance of blood glucose level in diabetes and treatment of liver and kidney ailments.

#### Plant Material:

Fresh *Cichorium intybus* Leaves were collected from chicory farm in Vaniyambadi, Tamilnadu, India and shade dried for 21 days. The dried leaves were finely powdered and kept in air-tight glass containers before use. The plant was authenticated by a botanist, Bangalore University, Bangalore.

#### Cichorium intybus:

(Dried leaf powder 500 mg/kg body weight- fed rats *(* for 10 days) were rendered diabetic and used 48 h after alloxanizatioun to demonstrate the neuroprotection by investigating AChE activity in the brain. For this, AChE isozymic spectrum was resolved by polyacrylamide gel electrophoresis and compared with that of control and diabetic pattern in the cerebral cortex.

50% (w/v) homogenates of the cerebral cortex were prepared in deionized distilled water and centrifuged at 7000 rpm for 1 h at 4°C. Ten μl of the supernatant was used for the electrophoretic run. The gels were run at 6 m Amps/tube (D.C. power supply) for 2 h using Tris-Boric acid-EDTA buffer at pH 8.5.

After the run, the gels were cut into 2 mm bits and each bit was homogenized in 1 ml of phosphate buffer (pH 7.0) centrifuged and used immediately for AChE determination as described above[11] and the pattern presented graphically.

## Results

The weight of the animal and its brain exhibited considerable decrease as a function of the disease [Table 1]. The blood sugar level demonstrated 120.5% elevation as a function of the disease [Table 1].

**Table 1:** Changes in glucose levels in blood of albino rats as a function of alloxan diabetes

Body wt (g)	Brain wt (mg)	Blood glucose (mg/100 ml)
Control		
80 ± 2.3	1.7 ± 0.08	102 ± 3.2
Diabetic		
75 ± 1.6	1.5 ± 6.0	225 ± 7.5
Percentage change		
–6.2	–4.9	+ 120.5
NS	NS	< 0.001

Values are mean ± SD of 26 observations

From the values given in Table 2, it is clear that the activity level of AChE was decreased in the cerebrum, cerebellum and medulla oblongata from diabetic rats. As the animals were experimented 48 h after alloxan administration, the observed decline in enzyme activity is related to early stage of alloxan diabetes. It is also seen [Table 2] that the decrease in the level of AChE activity showed variation in relation to the region of the brain. The activity level of AChE was higher in the cerebrum of both normal and alloxan diabetic rats compared to the other regions of the brain. Further, contrary to expectation, the cerebrum showed marked response for the change in the activity level of AChE and not the brain stem [Table 2].

**Table 2:** Activity levels of AChE in different regions of the brain of control and diabetic albino rats

Cerebrum	Cerebellum	Medulla oblongata
Control		
3.6 ± 0.9	1.77 ± 0.02	1.5 ± 0.01
Diabetic		
0.96 ± 0.01	1.2 ± 0.025	0.63 ± 0.02
*-73.32	*-32.21	*-57.4
*P*<0.001;	*P*<0.05	*P*<0.01

* Percentage decrease over control, (AChE activity expressed as milli moles ACh hydrolyzed/min/mg protein. Values are Mean ± SD of 9 observations)

## Discussion

The decrease in the activity level of AChE in diabetes observed may be a reflection of the type of electrical change in the nervous tissue on inducing alloxan diabetes. Such a decrease in the activity levels of AChE in different regions of the brain of alloxan diabetic rats may be either due to the inhibition of the enzyme synthesis by the altered cellular environment prevailing in the brain, or due to a decrease in the rate of enzyme synthesis. Earlier studies also indicated the inhibition of AChE synthesis in the CNS of rats with alloxan diabetes. It is suggested that the cause of inhibition of AChE synthesis is inadequate permeation of glucose across the surface membrane during diabetes.

**Figure 1:**
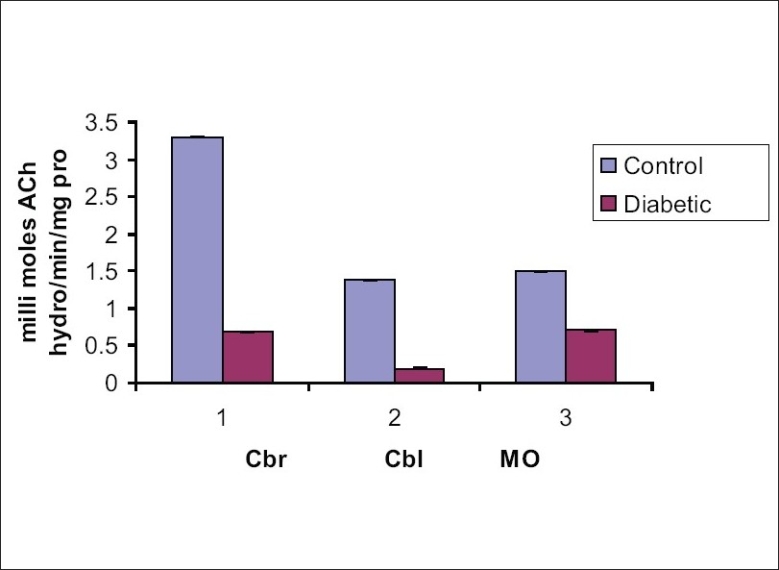
Effect of cerebral extract of diabetic rat on the AChE activity of the brain of control albino rats

Activity levels of AChE in the cerebrum, cerebellum and medulla oblongata of normal animals determined in the presence of the cerebral extract from diabetic animals were significantly low [Table 3, Figure 1]. The changes produced by the nervous extract of diabetic rats indicated the presence of an inhibiting substance/s in the cerebral extract of alloxan diabetic rats capable of depressing the AChE activity of normal animal on *in vitro* administration.

**Table 3:** Effect of cerebral extract of diabetic rat on the AChE in different regions of the control albino rats

Nature of the extract used	Cerebrum	Cerebellum	Medulla Oblongata
Normal cerebral extract			
Treated tissue (Controls)	3.30 ± 0.01	1.38 ± 0.02	1.50 ± 0.012
Diabetic cerebral extract			
Treated tissue (Exptls)	0.69 ± 0.01	0.09 ± 0.01	0.71 ± 0.025
Percentage Change	-79.1	-93.47	-46.0
(decrease)	*P*<0.001	*P*<0.01	*P*<0.01

[Activity is expressed as milli moles ACh hydrolyzed/min/mg protein. Values are Mean ± SD of 7 observations]

**Figure 2:**
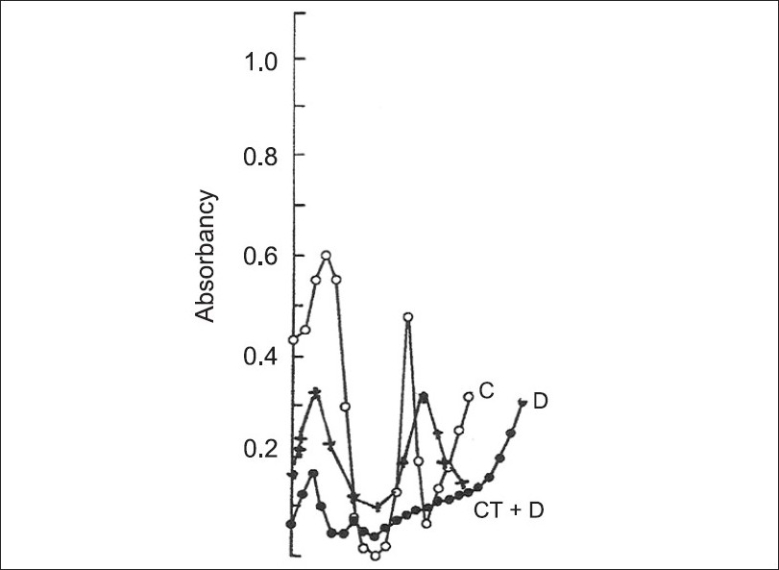
Densitometry of AChE separated on polyacrylamide gel electrophoresis in Raymond's buffer pH 8.5 C= Control 2 Iso; D=Diabetic 1 Iso; CT+D=Cichorium intybus treated Diabetic (2 isozymes restoration to normal level)

From the isozymic specrum presented in Fig 2, it is clear that the decline found in the activity of AChE is due to abolition of an isozymal form during diabetic state. Characteristically, in the diabetic rats (fed with dried leaf powder of *Cichorium intybus),* the isozymic pattern returned to the control (Gr 1) pattern with 2 isozymes. This restored neuronal efficiency and offered neuroprotection.
